# The burden of chickenpox disease in Sweden

**DOI:** 10.1186/s12879-016-1957-5

**Published:** 2016-11-10

**Authors:** Katarina Widgren, Johan Giesecke, Lars Lindquist, Anders Tegnell

**Affiliations:** 1 Department of Medicine, Huddinge, Karolinska Institutet, Stockholm, Sweden; 2 Department of Monitoring and Evaluation, The Public Health Agency of Sweden, Solna, Sweden; 3 Department of Medical Epidemiology and Biostatistics, Karolinska Institutet, Stockholm, Sweden; 4 Department of Infectious Diseases, Karolinska University Hospital, Stockholm, Sweden

**Keywords:** Chickenpox, Burden of Illness, Population register

## Abstract

**Background:**

Chickenpox vaccine is not included in the routine childhood vaccination programme in Sweden. The aim of this study was to estimate the baseline of national chickenpox disease burden, as comprehensive studies, required for an assessment regarding vaccine introduction, are lacking.

**Methods:**

We used available health care registers and databases; the death register, hospitalisations register, communicable disease notifications database, Stockholm County registers on consultations in specialist and primary care, temporary parental benefit to care for a sick child, and searches on the health care system’s website.

From each data source, records regarding chickenpox were identified and extracted, either using relevant diagnosis codes (ICD-10) or key words. A descriptive analysis with regards to number of cases and incidence, severity, and seasonality, was carried out covering the time period 2007 to 2013.

**Results:**

There were on average 333 patients hospitalised annually due to chickenpox, yielding a hospitalisation rate of 3.56/100,000 person-years. We found a slight male predominance in hospitalised cases. The highest hospitalisation rate was seen in 1 year-olds, whereas the peak in primary care consultations was in 2 year-olds. Nearly a quarter of children had parents who reported absence from work to care for them when sick with chickenpox. The average yearly death rate from chickenpox was 0.034/100,000 person-years. The duration of hospital stay increased with age. The seasonality in number of searches on the health care website corresponded well with hospitalisations and primary care consultations with peaks in spring.

**Conclusions:**

This study shows chickenpox death and hospitalisation rates in range with other European countries without routine vaccination. Swedish children fall ill with chickenpox at a very young age.

The study provides essential input for future discussions on the introduction of routine chickenpox vaccination in Sweden.

## Background

The varicella zoster virus (VZV), a herpesvirus with human as only known reservoir, is easily transmitted and thus, most people are infected already in childhood. VZV causes two distinct diseases; the primary manifestation chickenpox (varicella) is followed by life-long virus latency in the nervous system’s dorsal root ganglia and when reactivated can present with shingles (herpes zoster). The present study focuses on chickenpox, a well-known fever and rash illness. Chickenpox is generally considered a mild disease of children and most cases will not seek medical attention. However, severe complications are seen both among child and adult cases; mainly neurological involvement and secondary bacterial infections. The risk for severe disease increases with age but is also elevated in infants and the immunosuppressed [1, 2].

Since next to everybody acquires chickenpox in Western countries, the yearly incidence is approximately one birth cohort. However, the epidemiology varies between countries, even within Europe. Hospitalisation rates do not look entirely the same in register-based studies. Further, seroepidemiological studies show differences in age of infection with children acquiring antibodies earlier in Northern and Western Europe compared to Southern and Eastern, often explained by differences in child care. This leaves discrepancies in the percentage of the young adult population who are still seronegative [3, 4].

There are vaccines against chickenpox; currently all are live attenuated vaccines, considered safe and effective. Chickenpox vaccine is not included in the routine childhood vaccination programme in Sweden. In countries where it is, it has had a dramatic effect on disease burden. Both the World Health Organization (WHO) and the European Centre for Disease Prevention and Control (ECDC) acknowledge a favourable impact of routine vaccination as long as high coverage can be achieved [2, 3]. Further, they encourage countries to set up varicella surveillance and assess the epidemiology before deciding on or implementing routine vaccination, since the epidemiology will influence the need of a programme and should have bearing on its design and in the continuation its cost-effectiveness. In Sweden, there is no such up-to-date information on the epidemiology. Neither chickenpox nor shingles are notifiable diseases, except for their severe complication meningoencephalitis. In recent years, several studies on the national disease burden of shingles have been presented [5–7], whereas the national burden of chickenpox disease has only sporadically been assessed. In a seroepidemiological study using blood samples from 1997, Svahn et al. found approximately 98 % of 12 year-olds to be seropositive [8]. In a register-based assessment, Linde et al. found 322 children and 154 youths and adults were hospitalised in 1993 in Sweden due to chickenpox [9]. Grimheden et al. showed that the hospitalisation rate among Stockholm children was 1.6/1,000 chickenpox cases in 1998–2005 and only a minority (28 %) of these children had any underlying illness [10].

## Methods

In this retrospective study we used available data from a range of established health care registers and databases to make a comprehensive assessment of the chickenpox disease burden in Sweden in recent years.

Only data concerning chickenpox were extracted. To identify chickenpox-related records, we used either relevant key words or ICD-10 codes (International Statistical Classification of Diseases and Related Health Problems - Tenth Revision); B01.0 Chickenpox with meningitis, B01.1 Chickenpox encephalitis, B01.2 Chickenpox with pneumonia, B01.8 Chickenpox with other specified complications and B01.9 Chickenpox without complications.

### Data sources and data extraction

#### Deaths

The Cause of Death Register is kept by the National Board of Health and Welfare and holds data on causes of death for all Swedish citizens deceased in Sweden or abroad. Relevant records of chickenpox-related deaths were identified using ICD-10 codes.

#### Hospitalisations

The National Patient Register, also kept by the National Board of Health and Welfare, holds data on all hospital inpatient care. Reporting of hospitalisations are mandatory by law and the register has 99 % completeness. An evaluation has also shown high validity of the register [11]. Chickenpox-related records for this study were identified using the relevant ICD-10 codes; both records with chickenpox as primary and secondary diagnosis were extracted. Data on gender, age (in years) and relevant dates were extracted.

#### Consultations in specialist and primary care

Data on consultations in specialist and primary care were not available for the entire country.

The Stockholm Health Care Databases kept by the Stockholm County Council hold the same data as the National Patient Register, but restricted to hospitalisations within Stockholm County (≈2 million population). In addition, the registers hold data on consultations in specialist and primary care in the county (85–95 % coverage, personal communication Gunnar Ljunggren, Stockholm County Council). The same inclusion criteria and variables as above were used.

#### Notifications

The SmiNet2 database at the Public Health Agency holds notifications of communicable diseases. Cases with viral meningoencephalitis whose cerebrospinal fluid was found PCR-positive for virus or where intrathecal antibody production was detected, fall under mandatory reporting from both the diagnosing laboratory and the diagnosing doctor. The notification forms hold information on the relevant virus, age, gender and - with poor completeness - the clinical presentation, i.e. in this case whether the VZV-meningoencephalitis was related to chickenpox or to shingles.

#### Web searches

A project based at the Public Health Agency [12] has continuous access to anonymous free text searches on the health care system’s website (http://www.1177.se) and has previously been used for e.g. influenza surveillance. In this study, search terms including varicella, chickenpox, and “vattkoppor” (chickenpox in Swedish), along with various misspelt versions of these words were monitored.

This data source was included mainly with the objective to capture variations over time, as it was assumed to be timely and have high sensitivity to capture changes in incidence.

#### Temporary parental benefit when caring for sick child (VAB)

Through collaboration with the Swedish Social Insurance Agency, data on cause and length of parents’ absence from work to care for sick child were collected. Parents need to report VAB to the Social Insurance Agency on the first day of their absence from work in order to receive the temporary benefit. Symptoms of the child are probed for in the reporting form by a set of alternatives, including chickenpox. Recognition of this disease among Swedish parents has been shown to be high [13].

This data source was assumed to register a larger proportion of cases than the sources described above, as it captures also mild cases not seen by the health care system. Data was delivered aggregated on year. We included reports on children of 14 years and below.

#### Population data

Data on yearly average age-specific population size (age during the year) were taken from the website of Statistics Sweden (﻿http://www.scb.se).

### Study period

For each register/database we used data from as early as possible after January 2007 until as late as available. The period of available data differed somewhat between registers, but there was good overlap. From the health care registers the latest available data were extracted, i.e. the six year period January 2007 to December 2012. For the other data sources, all available data were included. Data on web searches and high quality data on VAB could only be retrieved﻿ for 2011–2013. We used notification data from 2007–2013.

### Data analysis plan

The entire analysis only included chickenpox-related records, as described above.

The majority of cases were children and data were analysed for each year of age separately from 0 to 9 years. Adolescents and adults were analysed by age groups: 10–14 years, 15–24 years, 25–44 years, 45–64 years, and 65 years and above.

For cases with more than one health care contact, only the first hospitalisat﻿ion, consultation in specialist or in primary care was used in relevant analyses.

All register data were taken at face value, no corrections or consistency checks were carried out. The rationale behind this was that if these data sources would constitute a basis for future routine chickenpox surveillance, limited time and resources would be available for data cleaning and management.

Data were analysed with regards to the three aspects; number of cases and incidence, severity, and seasonality.

#### Number of cases and incidence

We carried out a descriptive analysis of number of chickenpox cases and events, i.e. deaths, hospitalisations or consultations in specialist or primary health care (only for Stockholm County), overall and by age and gender, as well as the overall and age-specific incidence. Results are presented both for cases that had chickenpox as primary diagnosis and diagnosis in any position.

We investigated the number of chickenpox-afflicted children whose parents were absent from work and the age distribution of these children, the overall, age-specific and cumulative incidence.

#### Severity

The separate data sources capture a range of severity levels of disease; fatal cases and cases with notified meningoencephalitis were defined to represent severe disease.

Severity was also assessed by looking at complications according to the ICD-10 codes among hospitalised patients as well as length of hospital stay and VAB. The number of VAB days are presented as gross days (i.e. calendar days) the parents were absent from work.

#### Seasonality

The numbers of chickenpox cases by month by data source were described in a time-serie in order to explore seasonality. Periodograms were used to further investigate the seasonal pattern.

The correlation between monthly numbers of web searches and numbers of cases by the other data sources were analysed with negative binomial regression adjusting for number of web searches in previous months (lags).

## Results

### Number of cases and incidence

From 2007 to 2012 there were annually between 280 and 386 cases hospitalised with chickenpox in Sweden, with an average of 333 patients, or an average hospitalisation rate of 3.56/100,000 person-years. As some patients were admitted on several occasions, these patients yielded a total 377 hospitalisations and 1,740 hospital days per year. In addition, the incidence of making at least one consultation in specialist care was 20.1 patients/100,000 person-years and in primary care 109 patients/100,000 person-years, based on Stockholm County data (Table 1).

**Table 1:** Average yearly number of cases and average incidence of chickenpox in Sweden

	Primary diagnosis		Primary or secondary diagnosis	
	(*n*)	(per 100,000 pop.)	(*n*)	(per 100,000 pop.)
Deaths^a^	0	0	3.2	0.034
Hospitalisations^a^	256	2.74	378	4.04
Hospitalised patients^a^	236	2.52	333	3.56
Hospital days^a^	979		1740	
Consultations in specialist care^b^		19.7		22.9
Consultations at least once in specialist care^b^		17.6		20.1
Consultations in primary care^b^		112		118
Consultations at least once in primary care^b^		104		109
Parents absence from work to care for sick child, VAB gross days^c^	98730			
VAB number of children^c^	26072	1631		

^a^2007–2012, national data

^b^2007–2012, Stockholm County data, only incidence reported for comparability

^c^2011–2013, national data

The median age was 4 years among hospitalised cases and 3 years among those consulting any type of outpatient care. The peak incidence of hospitalisations was among 1 year-olds, and among 0 year-olds for consultations in specialist care. The VAB peak was among parents of 2 year-olds and also the peak of primary care visits in Stockholm was among 2 year-olds (Table 2).

**Table 2:** Average yearly number of hospitalised chickenpox cases, average length of hospital stay and average incidence of chickenpox-related health care contacts and parents’ absence from work to care for sick child (VAB), by age group, in Sweden, 2007-2012

Age (years)	Hospitalised patients (n)	Hospitalisation rate ^a^	Average length of hospital stay (days)	Specialist consultation rate ^a^, ^b^	Primary care consultation rate ^a^, ^b^	Parents absence from work to care for sick child, VAB rate ^a ^, ^c^
0	40.2	36.1	3.7	262	376	18
1	45.3	41.0	3.0	221	1204	3246
2	36.0	32.8	3.7	182	1325	4615
3	29.5	27.3	4.3	142	1146	4198
4	22.7	21.3	3.3	106	938	3861
5	20.8	19.8	3.9	87.0	708	2967
6	11.0	10.6	4.2	53.6	493	1922
7	10.3	10.3	9.3	38.﻿0	305	1123
8	5.2	5.1	10.2	22.1	199	644
9	4.3	4.4	20.6^d^	23.1	137	333
10–14	13.3	2.6	3.9	11.﻿9	70.8	54.9
15–24	18.5	1.5	8.2^d^	8.﻿8	34.7	
25–44	37.0	1.5	3.2	8.﻿5	28.6	
45–64	18.2	0.8	11.0	1.﻿5	8.﻿1	
65+	20.3	1.2	11.4	1.﻿7	4.﻿4	
Total	333	3.56	5.1	20.1	109	
Total children (≤14 years)	239	15.3	4.3	87.﻿6	518	1631

^a^ per 100,000 person-years in population of the same age

^b^ Stockholm C﻿ounty only

^c^ 2011-2013

^d^ one patient hospitalised for more than one year, according to record

Over the six year period, 55 % of hospitalised cases and 52 % of patients consulting in both specialist and primary care, were male. There was no statistically significant difference in the gender distribution over the age groups (*p* = 0.31, *p* = 0.28 and *p* = 0.59, respectively).

Cumulative incidence of chickenpox-related VAB – regardless of length of VAB - reached nearly to a quarter (23,196/100,000 person-years) of children below 15 years in Sweden.

### Severity

Over the 6 year period there was an average of 3.2 deaths/year (mortality rate 0.034/100,000 person-years), with a total of 19 deaths where chickenpox was contributing (*n* = 7) or underlying cause (*n* = 12). Chickenpox was not stated as the primary cause of death in any of the records. There was wide variation in primary cause of deaths and no interpretation as to its relation to the chickenpox illness can be made. The range of yearly deaths was 1 to 5 and the age range 1 to 91 years, with a median age of 58 years. Seven people were below 15 years. Among children 4 of 7 (57 %) deaths were girls and among adults 5 of 12 (42 %) were women.

The highest notification rate for VZV-meningoencephalitis was seen in the older age groups. For children below 10 years the incidence was less than 0.5 cases/100,000 person-years, whereas in all age groups above 10 years the incidence was greater than 0.5. During 2007–2013, clinical data were only reported for 146 of 686 notified cases (21 %). Of those 54 % lacked information on presence of rash and thus, making it  difficult to differentiate between chickenpox and shingles as the cause of meningoencephalitis.

**Fig. 1:**
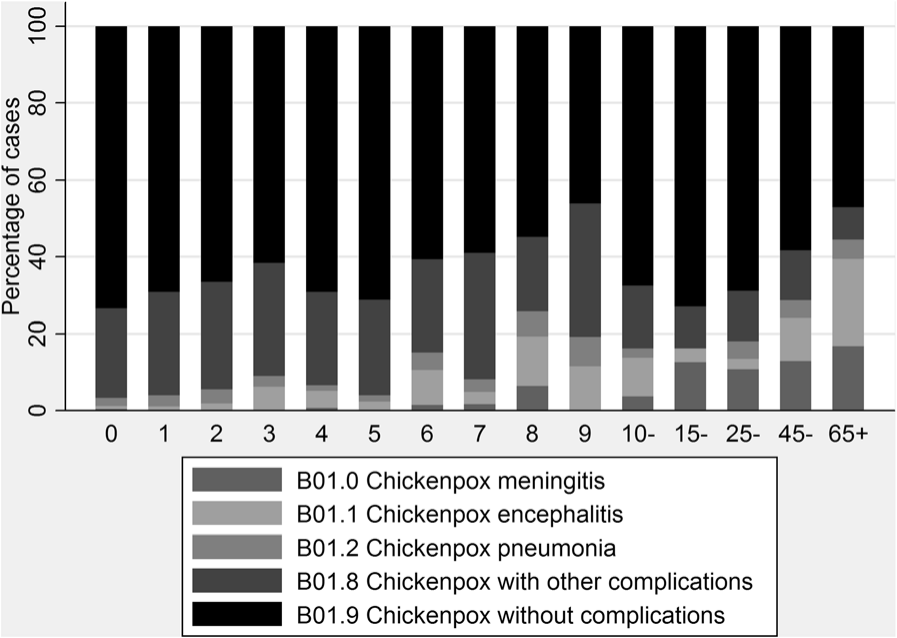
Frequencies of ICD-10 codes for chickenpox and its complications, by age group, among hospitalised chickenpox cases with a B01.x diagnosis in Sweden, 2007–2012

The percentage of admitted patients with ICD-10 codes indicating chickenpox with complications also increased slightly with age (*p* = 0.001). Chickenpox with encephalitis or meningitis was more frequent among adult cases, whereas other specified complications (B01.8) were seen more among the children (Fig. 1). The length of hospital stay increased with age (*p* < 0.001) (Table 2), whereas the length of VAB was similar for children of all ages, around 3.5 days.

### Seasonality

**Fig. 2:**
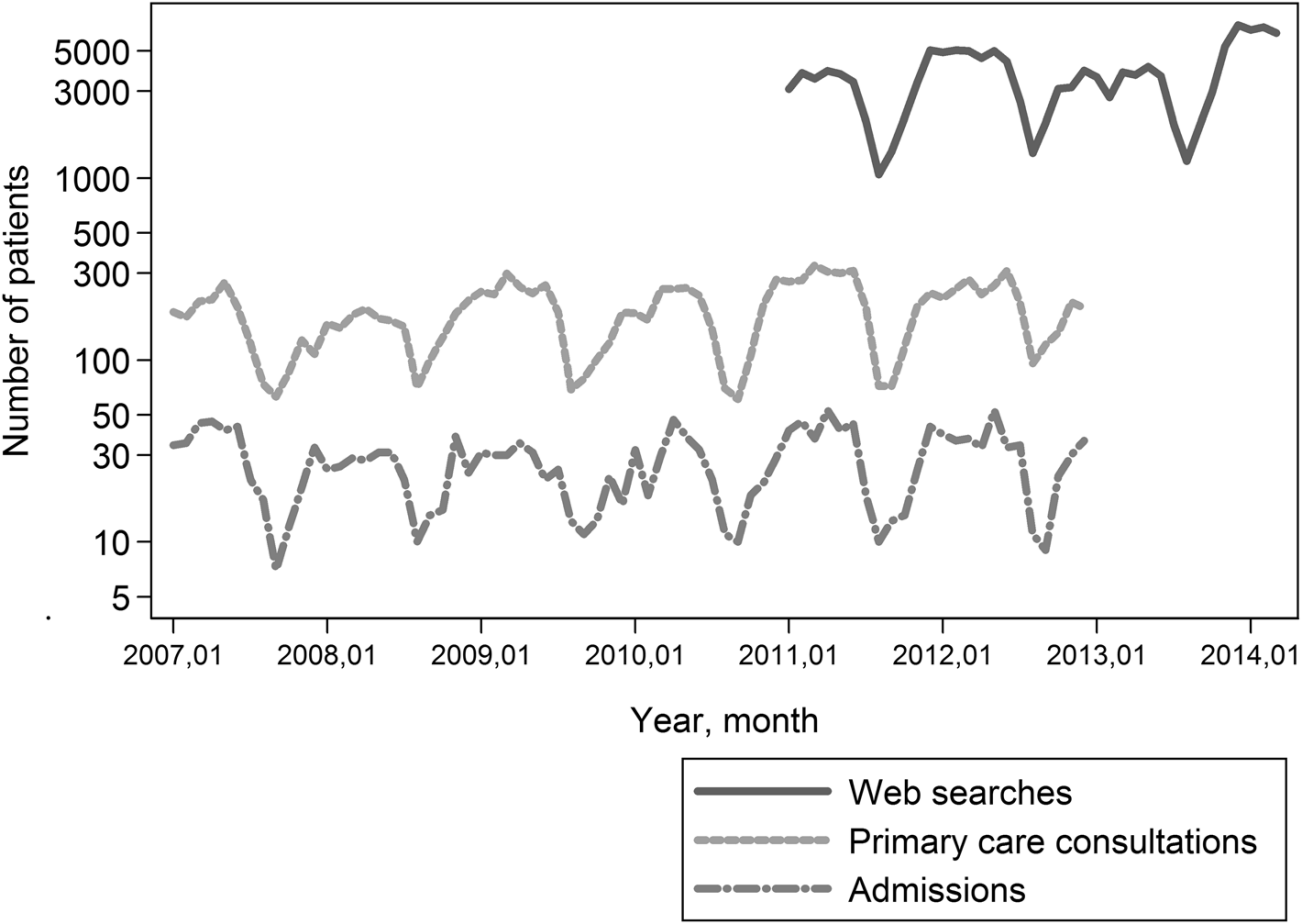
Number of chickenpox-related web searches, consultations in primary care (only Stockholm Cou﻿nty) and hospitalisations, by month, in Sweden

Chickenpox-related web searches, hospitalisations, and consultations in primary care (Stockholm County only) all showed a yearly pattern in the periodograms. As seen in Fig. 2, there were high numbers of cases during winters to spring. The lowest numbers were seen in August or September of each year. The seasonality pattern was mainly observed among young cases; no seasonal variation could be observed in the number of admitted adults above 45 years of age.

The number of web searches were associated with number of hospitalisations in the same month and with numbers of consultations in primary care in Stockholm in the same and the previous month.

## Discussion

This study was constructed to provide a baseline for chickenpox disease in Sweden before a decision regarding national routine vaccination.

Very few people died of the disease (0.034/100,000 person-years). The overall hospitalisation rate in Sweden over the study period (3.56/100,000 person-years) was in range with findings in other European countries without routine varicella vaccination, e.g. the Netherlands and Belgium, as well as Italy and Spain in the pre-vaccination era (1.9–8.9/100,000 person-years) [14–18]. With an assumed birth cohort (approximately 110,000) of chickenpox cases annually this rate corresponds to 3 hospitalisations/1,000 cases. The hospitalisation rate for ≤14 year-olds (15.3/100,000 person-years) was also comparable to international findings [17, 19], except for a higher rate in France [20].

We were anticipating a young age of infection in Sweden; high child care attendance from a young age has been correlated to early acquisition of chickenpox [4] and Swedish children have high participation rates in pre-school, over 85 % already at 2 years of age (the Swedish National Agency for Education, http://www.skolverket.se). We did find a majority of cases in children 5 years or below (58 % of all hospitalised cases), which is more than e.g. in Spain pre-vaccination [18]. There was a slight male predominance among hospitalised cases, also described elsewhere [19, 21].

The incidence of consulting specialist or primary care was 20.1 and 109 cases/100,000 person-years, respectively. The consultation rate in primary care was less than half of those in other countries [14–16], which is also seen in overall national comparisons and possibly explained by health care organization [22].

In previous studies [14, 18–21] the highest chickenpox hospitalisation rates were seen in children below 1 year (range: 39.2-149/100,000 person-years). Infants are known to have a more severe disease, they might also be admitted more generously than older children. Hospitalisation rates for 1–4 year-olds was slightly lower (30.7/100,000 person-years in our material) than in infants, similarly to other countries. However, when we split up this age group into yearly intervals we found the true peak hospitalisation rate in 1 year-olds (41/100,000 person-years) (Table 2). A Belgian study that did not group the youngest children also found a peak in 1 year-olds [16] and a Dutch study showed a peak in General Practitioner consultations in 1 year-olds [15]. It seems important that studies on age distribution of chickenpox report their findings for the youngest children in sufficient detail.

Increasing age is a known risk factor for severe chickenpox disease. In this study we found longer hospital stays and a somewhat larger proportion of ICD-10 codes for complicated chickenpox disease with increasing age. Also, the median age was slightly higher in hospitalised cases than patients seen in outpatient care, implying a higher age among severe cases.

Data from health care registers provide information on severe cases, which unquestionably are those a vaccination programme sets out to prevent and thereby are most important to monitor. However, in order to accurately describe the epidemiology, also mild cases need to be defined. Since age is an important risk factor for severe chickenpox disease it cannot be assumed that the age distribution of mild and severe cases look the same. We found most cases of severe disease in the young ages, where the complication rate is known to be at its lowest [23]. Thus, we suspect that a majority of chickenpox cases, also mild cases, fall ill at this early age. This is a desirable equilibrium when this disease is present and needs to be taken into account when assessing a vaccination programme. The best method to more precisely describe the age-specific incidence and capture also those who have had a mild disease and to determine the immunity in the population is a seroepidemiological study. Since this is a laborious, costly and also ethically sensitive issue as it involves drawing blood from healthy children, such a study has not been carried out for nearly two decades in Sweden. Instead we used a proxy for mild disease; temporary parental benefit when caring for a child sick with chickenpox. In fact, parents of more than 23 % of Swedish children were found here, i.e. one quarter of all children below 15 years have had a parent staying home from work because of chickenpox in the child. However, there were some data limitations; although a majority of parents were likely to be employed and fall under mandatory reporting of absence from work when caring for a sick child, reporting of the child’s symptoms/disease is not mandatory and there might be loss of information. Furthermore, there is no need to report sickness of child during the first 18 months of parental leave before a child starts day care and the data are truncated at around that age. Additionally, if a parent is on the 18 months parental leave for a younger sibling when an older sibling falls ill, there is no need for additional reporting of absence from work. Yet, parental benefit is a rather unique data source and the best we can do at the current time to define the age distribution of mild cases. Considering the limitations mentioned, the true peak incidence of mild disease should not be older than the 2 years found here; corroborating our assumptions of a young age of infection in Sweden. This data source also gives us an indication of the duration of disease in children (i.e. an average parental absence of 3.5 days).

Another way to capture mild disease was to include chickenpox-related searches on the health care system’s website. The main objective was to catch changes in seasonality. As previously described for chickenpox, the peak incidence was in the late winter to spring [24]. Seasonal variations are only seen for children, adult cases were fewer and more sporadic. There was strong correlation between the monthly number of web searches and the number of hospitalisations and consultations in primary care, respectively. Thus, monitoring of chickenpox web searches gives accurate information on the circulation of chickenpox without any reporting delay.

This study only captures cases with a record of chickenpox disease. Cases with late complications might be underestimated; e.g. there is increasing evidence of an elevated risk of stroke and other cerebrovascular insults in the wake of a chickenpox infection [25]. Even though, these complications are rare, the severity of each case will make a considerable impact on disease burden.

Except for taking all data at face value, without any validity checks, the accuracy of our results could be influenced by people getting chickenpox vaccination in the absence of routine vaccination. A number of people choose to get the vaccination at their own cost and in addition, susceptibles in the immediate family of particularly sensitive/ immunosuppressed patients are vaccinated against e.g. chickenpox (cocooning). In total roughly 1,000 chickenpox vaccinations are given yearly in Sweden (according to prescriptions in the Concise database), i.e. less than 1 % of a birth cohort or less than 0.5 % if two doses are given, which could not affect the results in any substantial way.

## Conclusion

The burden of chickenpox disease in Sweden is not negligible; the disease is common and sometimes severe. Swedish children fall ill with chickenpox at an early age. This study provides a baseline for the national disease burden and should serve as input for future discussions on the introduction of routine vaccination. The main limitation with these data is the difficulty in attaining reliable mild disease estimates.

## Abbreviations

ECDC: European Centre for Disease Prevention and Control
ICD-10: International Statistical Classification of Diseases and Related Health Problems - Tenth Revision
VAB: Temporary parental benefit when caring for sick child [Vård av barn]
VZV: Varicella zoster virus
WHO: World Health Organization
